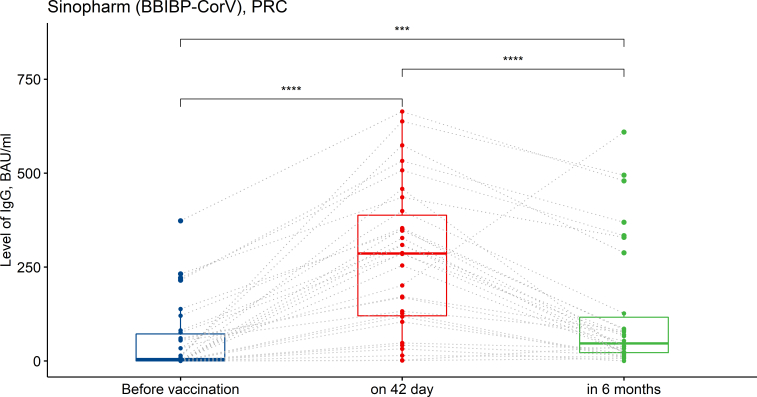# Comparative immunogenicity and safety of Gam-COVID-Vac and Sinopharm BBIBP-CorV vaccines: results of a pilot clinical study

**DOI:** 10.1016/j.heliyon.2023.e21877

**Published:** 2023-11-07

**Authors:** Igor Stoma, Katsiaryna Korsak, Evgenii Voropaev, Olga Osipkina, Aleksey Kovalev

**Affiliations:** Gomel state medical university, Gomel, Belarus

**Keywords:** SARS-CoV-2 infection, Vaccines, Humoral immune response, Enzyme-linked immunosorbent assay (ELISA), BAU/ml

## Abstract

**Introduction:**

There are few comparative studies on efficiency of broad range COVID19 vaccination strategy. This pilot aims to describe the effect of mixed COVID19 vaccination on vaccination adoption and subsequent total immunity, Conducted in Republic of Belarus, this pilot clinical study shows varying immunogenic responses to Sputnik V (Gam-COVID-Vac), Russian Federation (RF) and Sinopharm (BBIBP-CorV), People's Republic of China (PRC) vaccines.

**Objective:**

To compare the immunogenicity and reactogenicity of Sputnik V (Gam-COVID-Vac) and Sinopharm (BBIBP-CorV) vaccines in vaccinated individuals.

Materials and MethodsA total of 60 adults participated in the present study. The immune response after vaccination was assessed using enzyme immunoassay. IgG levels were measured in all participants at three time points: before vaccination, on the 42nd day after the first vaccine dose, and in 6 months after the first vaccine dose. Age, sex of participants, vaccine type, history of COVID-19/IgG seropositivity were included in the multivariate analysis. The results of the SARS-CoV-2 infection antibody test were quantified according to the WHO First International Standard (NIBSC code:20/136) and measured in international units (BAU/ml).

**Results:**

The study participants (n = 60) were divided into two groups where 50 % (n = 30) were vaccinated with Sputnik V (Gam-COVID-Vac), and 50 % (n = 30) were vaccinated with Sinopharm (BBIBP-CorV). Women represented 63 % and 77 % of Sputnik V and Sinopharm groups, respectively. The IgG levels on day 42 after the first vaccine dose were: Sputnik V (Gam-COVID-Vac): Me = 650.4 (642.2–669.4); Sinopharm (BBIBP-CorV: Me = 376.5 (290.9–526.4) (U_Mann–Whitney_ = 164, p = 0.000024). The IgG levels in 6 months after the first vaccine dose were: Sputnik V (Gam-COVID-Vac)Me = 608.7 (574.6–647.1); Sinopharm (BBIBP-CorV) Me = 106.3 (78.21–332.4); (U_Mann–Whitney_ = 172.5, p-value = 0.000042)). In a multivariate model Sputnik V vaccine type and IgG seropositivity at the baseline were significantly associated with higher levels of IgG both at 42 days and 6 months post-vaccination. Reactions after vaccination appeared in 27 vaccinated people (45 %).

**Conclusion:**

This pilot study demonstrated that Sputnik V (Gam-COVID-Vac) vaccine was more immunogenic than Sinopharm (BBIBP-CorV) vaccine. IgG levels in vaccinated individuals who previously recovered from SARS-CoV-2 infection (hybrid immunity) were higher than in SARS-CoV-2 infection immune-naive people. Reactions after vaccines administration were mild to moderate.

## 1 Introduction

The pandemic caused by SARS-CoV-2 infection has been ongoing for more than 3 years. Due to the lack of etiotropic therapy for COVID-19 [1], increased infectivity [2] and the acquisition of vaccine-resistant mutations of SARS-CoV-2 [3], vaccination is one of the few effective means of controlling the pandemic. Other measures implemented at varying degrees across the world include social distancing and masking, which have contributed to curbing the rates of transmission. Effective vaccination has a potential to curb the spread of the virus [4], as well as to reduce the consequences of infection and the burden on health care facilities [5]; effective pandemic control requires a high vaccination rate [6].

Up to January 2023, 50 vaccines against SARS-CoV-2 infection have been approved for mass administration worldwide [7]. They differ in their manufacturing technology, mechanism of action, and efficacy.

During the study period (2021–2022 years), only two vaccines were available for mass vaccination in the Republic of Belarus: Sputnik V (Gam-COVID-Vac), manufactured in the Russian Federation (RF) and Sinopharm (BBIBP-CorV), manufactured in People's Republic of China (PRC). As of December 15th, 2022, 70.7 % of population were vaccinated with two doses, 71.8 % received one dose of vaccine, and among them 80.7 % were booster vaccinated [8]. There are few published studies that directly compare immunological efficacy and postvaccination adverse events rates between Sputnik V (Gam-COVID-Vac) and Sinopharm (BBIBP-CorV) vaccines [[9], [10], [11], [12], [13]]. Additionally, there are studies dedicated to understand the Sputnik V immunogenicity characteristics [[14], [15], [16]] and some studies showing this data for Sinopharm vaccine [[17], [18], [19], [20], [21], [22], [23]]. Although vaccination supply remained high in the Republic of Belarus, scarcity of verified comparative information about available vaccines became a driver of distrust in the vaccination strategy. General population's reluctance to participate in the vaccination efforts contributed to Belarus' inability to achieve required vaccination coverage by a fast track for successfully curbing of the pandemic in the country.

## 2 Objective

Comparative description of immunogenicity and reactogenicity of Sputnik V (Gam-COVID-Vac), RF and Sinopharm (BBIBP-CorV), PRC vaccines among vaccinated people.

## 3 Materials and methods

### 3.1 Study design and participants

This clinical pilot study was carried out from October 2020 to December 2021 at Gomel State Medical University, Belarus. The majority of study participants were female (70 %). All study participants were divided into two groups, where 50 % were fully vaccinated with Sputnik V (Gam-COVID-Vac), among them 37 % were male and 63 % were female. Another group (50 %) were vaccinated with Sinopharm (BBIBP-CorV) vaccine, among them 23 % were male and 77 % were female. We took into consideration the following inclusion criteria: enrolled participants aged over 18 y.o and older; their consent to periodic check points by phone and home visits; informed consent form signed by a participant. Exclusion criteria were: pregnancy, breastfeeding, congenital or acquired immunosuppressive conditions, possible or confirmed case of COVID-19 on the day of vaccination. Blood sampling was taken 3 times: immediately before the first dose of vaccine, on the 42nd day, and in 6 months after the first dose of vaccine. All study participants were informed about the study design and upcoming procedures. The informed written consent for this study was collected by the authors and signed by all participants. The study received an approval from the Institutional Ethics Committee of Gomel State Medical University (protocol №3 from November 06, 2020). Full vaccination status was recorded when two doses of vaccines were administered to all of the participants. Vaccination was administered in adherence to manufacturer instructions and in accordance with principles of Good Distribution Practice (GDP). The interval between the first and the second dose was established as 21 days, in accordance to the instructions for both vaccines. Participants were assigned to the Sputnik V (Gam-COVID-Vac) or Sinopharm (BBIBP-CorV) group according to their personal choice of vaccine. No recommendations were given in choosing any of the provided vaccines at the stage of sampling. Package instructions as well as general information about vaccination were provided to all participants. No randomization was performed, but all participants’ characteristics were taken into account later at the analysis stage. There was a single blinding point in the study: the laboratory staff was specifically blinded at the stage of immunogenicity assessment. In case of a missed visit due to non-medical reasons, a participant was excluded from the study. On every visit, participants completed a questionnaire including data on the course of chronic diseases, history of COVID-19, and adverse reactions if any. In case of any adverse reactions due to vaccination the principal investigator was informed immediately. The double-controlled system of data collection was used with an additional checking of database by the non-affiliated supervisor. Electronic database was anonymized prior to statistical processing. Potential bias was limited due to the inaccurate data reporting by the participants.

### 3.2 Laboratory techniques

The study utilized participants' plasma collected according to the standard protocol and kept at −20 °C. Immune response to vaccination was evaluated by performing by enzyme immunoassay using the Sunrise Tecan Microplate Photometer (Austria). A reagent kit manufactured by Vector-Best (Russian Federation) and designed for enzyme immunoassay for quantitative detection of SARS-CoV-2 class G immunoglobulins, SARS-CoV-2-IgG quantitative ELISA-BEST was used for the immunoassay. According to the manufacturer's instructions, this kit is suitable for the quantitative detection of specific IgG in SARS-CoV-2 in infected and re-vaccinated participants. It can also be used in evaluation of post-vaccination immune response during immunization with vaccines based on different technologies, including vector vaccines, mRNA and inactivated whole-virion vaccines. The SARS-CoV-2-IgG quantitative ELISA-BEST reagent kit design uses recombinant full-length trimerized S glycoprotein (Spike) of the SARS-CoV-2 virus derived from an eukaryotic expression system. The protein molecule consists of two subunits: S1 containing the RBD domain and S2. The SARS-CoV-2-IgG reagent kit quantitative ELISA-BEST detects the pool of immunoglobulin class G synthesized to all antigenic determinants of protein S including the RBD-domain. These test systems are widely used, checked for cross-reactivity, officially evaluated and licensed in Eastern Europe and Asia [[24], [25], [26]]. Performance of this kit was previously compared with Anti-SARS-CoV-2 ELISA (IgG) kit (Euroimmun, Germany) [27].

The quantification of the SARS-CoV-2 infection antibody assay is based on the WHO First International Standard (NIBSC code:20/136) and measured in international units (BAU/ml).

According to the manufacturer's instructions, the diagnostic sensitivity to the detection of IgG to SARS-CoV-2 is 100 % (range 95.7%–100 %, 95 % confidence interval). The diagnostic specificity is 100 % (range 98.5%–100 %, 95 % confidence interval). The information letter accompanying the reagent kit SARS-CoV-2-IgG Quantitative ELISA-BEST, No. RZN 2021/14458. The kit reports that virus neutralizing activity with a neutralization titer of 1/160 or higher is observed in all samples with a specific IgG concentration of 150 BAU/ml or higher (ELISA titer ≥1/600; 95 % CI: 83.16–100 %) and only 50 % of samples showed a specific IgG concentration of 80–149 BAU/ml (ELISA titer ≥1/400 - 1/800; 95 % CI: 32.43–67.57 %), a specific IgG level of less than 10 BAU/ml should be considered as a negative result of the quantitative assay.

### 3.3 Statistics

Statistical processing of the results was performed using the R Statistical Programming Environment (R version 4.1.1; R packages: ggpubr_0.4.0, ggplot2_3.3.5, dplyr_1.0.7). Quantitative comparisons of linked samples, i.e. changes in the IgG levels over time in groups with different vaccines, were performed using the Friedman test followed by pairwise comparison of groups the Wilcoxon test with Bonferroni multiple comparison adjustment. Mann-Whitney test was used for unrelated samples to compare the dynamics of IgG levels in the group with Sputnik V (Gam-COVID-Vac) to the group with Sinopharm (BBIBP-CorV). A qualitative comparison of the groups (obtained of one of the IgG values: up to 150 BAU/ml, 150–500 BAU/ml, >500 BAU/ml after administration of Sputnik V and Sinopharm vaccines; incidence of major postvaccination reactions among participants) was evaluated using the Pearson χ2 test. Fisher's Exact Test was used in cases where expected values in the cells of the contingency table were less than 5. Due to the limited sample size and a double-controlled system of data collection there was no cases of missing data at the statistical analysis stage. The significance level was set at 0.05. Multiple linear regression model included age, sex, vaccine type and history of COVID-19/IgG positivity as variables in the analysis, which was performed for each endpoint (42 days, 6 months).

## 4 Results

This pilot study originally enrolled 89 participants; 29 dropped out due to external circumstances and the final cohort included 60 participants. (Fig. 1).

**Fig. 1:**
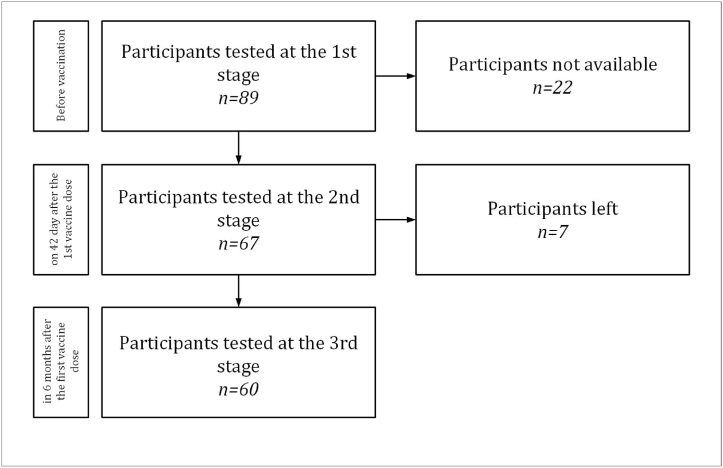
Flowchart of the study.

According to the questionnaire, 72 % of participants reported having no chronic diseases. The indicated chronic diseases were reported as follows: 1) cardiovascular diseases by 35 % of all participants with chronic diseases, 2) gastrointestinal diseases by 41 %, 3) musculoskeletal diseases by 18 %, 4) respiratory diseases by 12 %, 5) endocrine diseases by 6 %, 6) female reproductive system diseases by 6 %. Two participants reported having more than one chronic disease. When comparing the two groups of participants on the basis of chronic diseases, age, gender and COVID-19 history, no significant difference was found at the baseline (p > 0.05). Therefore, the studied 2 groups of participants were actually matched by those covariables at the baseline. The characteristics of the study participants are presented in Table 1.

**Table 1:** The demographic and clinical characteristics of the study participants (N = 60).

	Sinopharm (BBIBP-CorV), PRC	Sputnik V (Gam-COVID-Vac), RF	Total
	(N = 30)	(N = 30)	(N = 60)
**Age (median, IQR)**	44.5 [37.25, 49.75]	47.5 [40.75, 59.00]	46.0 [39.00, 57.75]
**Gender:**			
female	23 (76.7 %)	19 (63.3 %)	42 (70.0 %)
male	7 (23.3 %)	11 (36.7 %)	18 (30.0 %)
**Pre-vaccination COVID-19 history:**			
yes	11 (36.7 %)	18 (60.0 %)	29 (48.3 %)
no	19 (63.3 %)	12 (40.0 %)	31 (51.7 %)
**Chronic diseases:**	8 (47.1 %)	9 (52.9 %)	17 (100.0 %)
Cardiovascular	2 (25.0 %)	4 (44.4 %)	6 (35.3 %)
Gastrointestinal	4 (50.0 %)	3 (33.3 %)	7 (41.2 %)
Musculoskeletal	2 (25.0 %)	1 (11.1 %)	3 (17.6 %)
Respiratory	0 (0 %)	2 (22.2 %)	2 (11.8 %)
Endocrine	0 (0 %)	1 (11.1 %)	1 (5.9 %)
Female reproductive	0 (0 %)	1 (11.1 %)	1 (5.9 %)

**Fig. 2:**
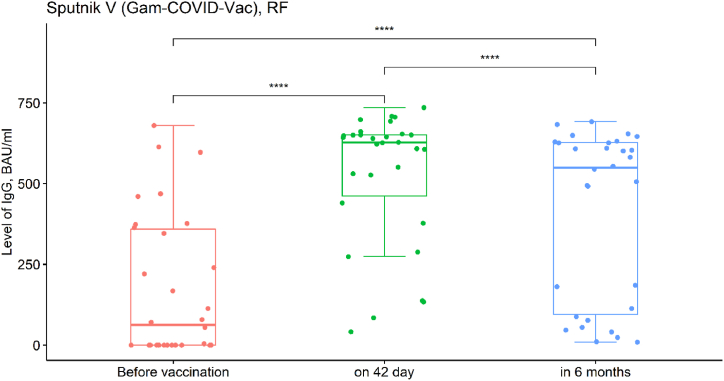
Dynamics of Ig G production in response to Vaccine Sputnik V (Gam-COVID-Vac). (before vaccination - Ig G value (BAU/ml); on 42 day - Ig G value (BAU/ml); in 6 months after the first vaccine dose - Ig G value (BAU/ml). Individually matched data is shown in Suppl., SF_2.

**Fig. 3:**
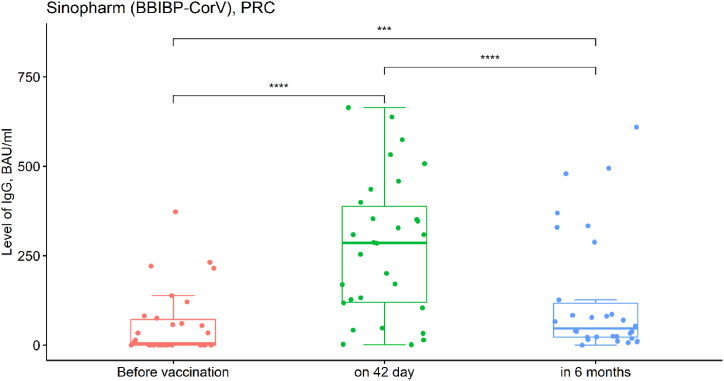
Dynamics of IgG appearance in response to Sinopharm (BBIBP-CorV) vaccine (before vaccination - IgG value (BAU/ml); on 42 day after the first dose - IgG value (BAU/ml); in 6 months after the first vaccine dose - IgG value (BAU/ml). Individually matched data is shown in Suppl., SF_3.

**Fig. 4:**
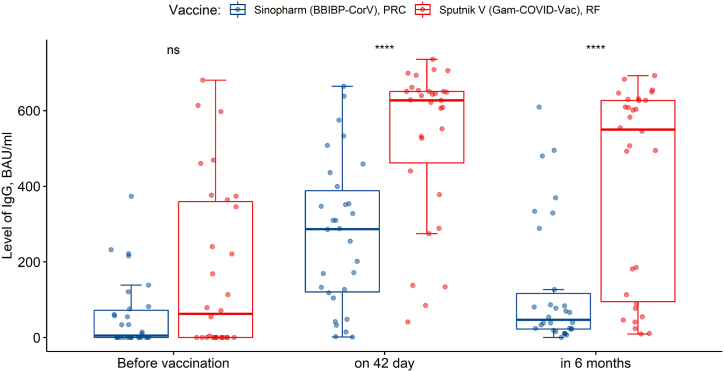
Comparison of the dynamics of IgG levels in response to the administration of Sputnik V (Gam-COVID-Vac) and Sinopharm (BBIBP-CorV) vaccine (before vaccination - IgG value (BAU/ml); on 42 day after the first dose - IgG value (BAU/ml); in 6 months after the first vaccine dose - IgG value (BAU/ml).

Immunogenicity is one of the proxy markers for a vaccine efficacy. In this study IgG levels to SARS-CoV-2 S-protein were tested in all participants at three stages: before vaccination, on 42 day after the first dose of vaccine and in 6 months after the first dose of vaccine. The unit of measurement for IgG levels is BAU/ml. (Note: BAU-binding antibody units). The study data is shown in Fig. 2, Fig. 3, Fig. 4.

In Fig. 2, we demonstrate that quantitative IgG levels at three measurement stages have significant differences (Friedman chi-squared = 47.267, df = 2, p-value <0.001). IgG levels on the 42nd day were significantly higher compared to the before vaccination stage (T_Wilcoxon_ = 461, p_Bonferroni corr._ < 0.001). Comparing 42nd day and 6 months post vaccination, IgG levels demonstrated a significant drop (T_Wilcoxon_ = 447, p_*Bonferroni corr.*_ < 0.001); nevertheless, IgG level remained significantly higher than at before vaccination stage. (T_Wilcoxon_ = 452, p_*Bonferroni corr.*_ < 0.001).

As shown in Fig. 3, the quantitative IgG values at the three study stages are significantly different (Friedman chi-squared = 44.891, df = 2, p-value <0.001). Levels of IgG on the 42nd day after the first dose of vaccine were significantly higher than prior to vaccination (T_*Wilcoxon*_ = 465, p_*Bonferroni corr.*_ < 0.001)). In 6 months after the first dose, there was a significant decrease in IgG values (T_*Wilcoxon*_ = 428, p_*Bonferroni corr.*_ < 0.001), but they remained at a higher level compared to the before vaccination stage (T_*Wilcoxon*_ = 385, p_*Bonferroni corr.*_ < 0.001)).

At the pre-vaccination stage there is no significant difference between the Sputnik V (Gam-COVID-Vac) and Sinopharm (BBIBP-CorV) vaccines (U_Mann–Whitney_ = 349.5, p = 0.12). On the 42nd day after the administration of both vaccines the antibody levels increase, but the quantitative IgG value for the Sputnik V (Gam-COVID-Vac) vaccine is significantly higher (U_Mann–Whitney_ = 164, p < 0.001). This trend was also observed in 6 months after the first dose of both vaccines (U_Mann–Whitney_ = 172.5, p-value <0.001).

According to the questionnaire survey and the answers of study participants, 52 % of participants did not have COVID-19 before vaccination; 48 % had had the infection prior to vaccination. Having compared the groups’ characteristics on the basis of prior infection with SARS-CoV-2, no significant difference was found (p = 0.071).

As the factor of previous SARS-CoV-2 infection influences the outcome of the study, it was also included in the statistical analysis.

**Fig. 5:**
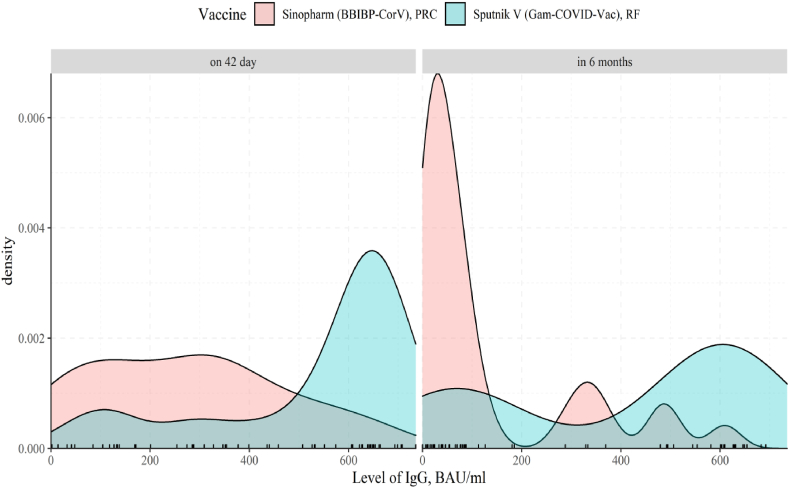
Comparison of the groups (vaccinated with Sputnik V (Gam-COVID-Vac) and Sinopharm (BBIBP-CorV)) by IgG levels (BAU/ml) – on day 42 and in 6 months after the first vaccine dose.

Fig. 5 shows that in the Sputnik V (Gam-COVID-Vac) vaccine group, the largest group of participants at the 42nd day stage had IgG levels above 500 BAU/ml, while most Sinopharm (BBIBP-CorV)-vaccinated patients had levels ≤500 BAU/ml (χ^2^ = 19.644; df = 2; p < 0.001). At the 6-months stage this trend continued: in the Sputnik V (Gam-COVID-Vac) vaccine group most participants had IgG levels above 500 BAU/ml, while most patients vaccinated with Sinopharm vaccine had IgG level below 150 BAU/ml (χ^2^ = 20.747; df = 2; p < 0.001). The groups were also compared taking into account the serological status of the study participants.

**Fig. 6:**
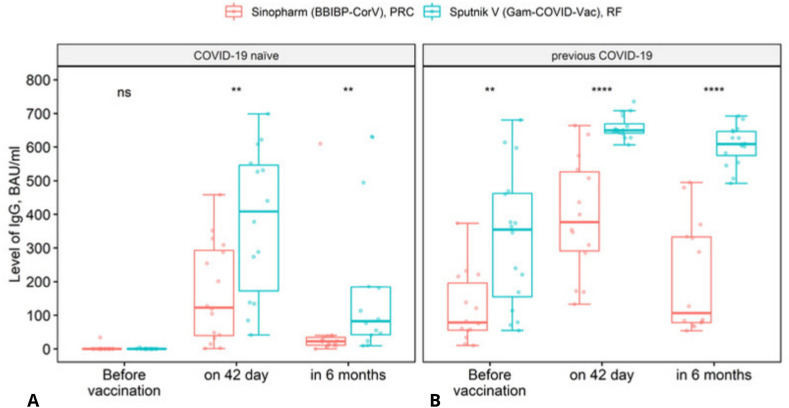
Comparison of Sputnik V (Gam-COVID-Vac) and Sinopharm (BBIBP-CorV) groups based on serological status before vaccination. Panel A: COVID-19 naϊve - COVID-19 – immune-naive people; panel B: previous COVID-19 – participants with previous COVID-19. Before vaccination – pre-vaccination IgG level; on 42 day – IgG level on 42 day after the first dose of one of the vaccines; in 6 months – IgG level in 6 months after administration of one of the vaccines.

Fig. 6 compares IgG levels to the SARS-CoV-2 between those with prior COVID-19 infection and COVID-19 naïve participants in both Sputnik V and Sinopharm groups. The highest antibody levels were observed on the 42nd day after vaccination in both the COVID-19 – immune-naive participants (U_Mann–Whitney_ = 47, p = 0.006) and previous COVID-19 groups (U_Mann–Whitney_ = 15, p < 0.001), in 6 months after vaccination the IgG value declined in those who had not previously been ill (U_Mann–Whitney_ = 42, p = 0.003) and in those who had had COVID-19 before vaccination (U_Mann–Whitney_ = 1, p < 0.001). However, the combination of antibodies generated by previous COVID-19 and the Sputnik V vaccine has higher titers and duration (U_Mann–Whitney_ = 1, p < 0.001).

**Fig. 7:**
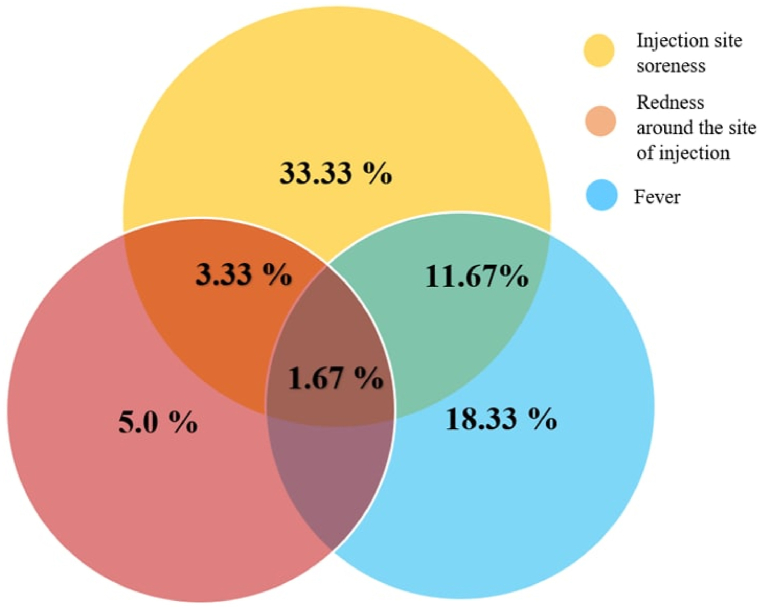
The main post-vaccination adverse event rate (and their combination) noted in the study participants: soreness and redness at the injection site, increased body temperature.

The rate of adverse events of Sputnik V (Gam-COVID-Vac) and Sinopharm (BBIBP-CorV) vaccines is worth considering separately, as this is a frequent topic of discussion. The incidence of post-vaccine reactions in this study was 45 %. All of the observed reactions were mild to moderate in severity. The most frequent ones were: injection site soreness (33.33 %), redness around the site of injection (5 %), fever (11.67 %) and a combination of these reactions (Fig. 7). Participants also reported weakness (1.67 %), headache (1.67 %), and swelling at the injection site (1.67 %).

Post-vaccination reactions were observed in both vaccinated groups (p = 0.119). However, significant statistical differences were found only regarding to the increase in body temperature; although in the group vaccinated with Sputnik V (Gam-COVID-Vac) this reaction was more frequent (p = 0.006).

## 5 Discussion

### 5.1 Implications for future research

Obtained study results may be generalized towards regions, where both vector vaccines and inactivated vaccines are widely used, especially in a condition of average vaccination coverage.

### 5.2 Results of multivariate analysis

Age, sex, vaccine type and history of COVID-19/IgG positivity were included as variables in a multiple linear regression model. This analysis was performed for each endpoint in the study (42 days, 6 months).

For the endpoint at 42 days: age was not associated with IgG levels (p = 0.441); sex was not associated with IgG levels (p = 0.66); type of vaccine had a significant association with IgG levels (p < 0.001). For Sputnik V (Gam-COVID-Vac) IgG levels were significantly higher than for Sinopharm (BBIBP-CorV) with an Estimate = 243.09. IgG seropositivity at the baseline (SP) was also significantly associated with higher IgG levels (p < 0.001), with a strong influence observed in the regression model (Estimate = 256.56). For the endpoint at 6 months: age was not associated with IgG levels (p = 0.887); sex was not associated with IgG levels (p = 0.551); type of vaccine had a significant association with IgG levels (p < 0.001). For Sputnik V (Gam-COVID-Vac) IgG levels were significantly higher than for Sinopharm (BBIBP-CorV); Estimate = 258.72. IgG seropositivity at the baseline (SP) was significantly associated with higher IgG levels (p < 0.001), with a strong influence in the regression model (Estimate = 286.81).

The level of the variance explained by the model (adjusted R-squared) was 0.5343 (42 days) and 0.5381 (6 months). These values were not high, probably due to some potential outliers in IgG levels.

### 5.3 Meaning of the study/understanding possible mechanism

A retrospective observational study conducted in Mongolia found the immunogenicity of two doses of Sputnik V (Gam-COVID-Vac) vaccine to be significantly higher than the immunogenicity of two doses of Sinopharm vaccine. No reactogenicity study was conducted in this study [28]. A retrospective observational study, conducted in Hungary, investigated the efficacy of Sputnik V (Gam-COVID-Vac) and Sinopharm (BBIBP-CorV) vaccines against SARS-CoV-2 infection with confirmed by PCR test and death cases from COVID-19 among vaccinated people [29].

The results of this study indicated that the Sputnik V (Gam-COVID-Vac) vaccine was 85.7 % effective against SARS-CoV-2 infection and 95.4–100 % effective against COVID-19 deaths (depending on age cohort), and the Sinopharm (BBIBP-CorV) vaccine was 68.7 % effective against SARS-CoV-2 infection and 67.3–100 % effective against COVID-19 deaths (depending on age cohort) [29].

Only few international publications reflect the results of a direct comparison of the reactogenicity of Sputnik V (Gam-COVID-Vac) and Sinopharm (BBIBP-CorV) vaccines. Based on a survey of vaccines the study, conducted in Iran, found that reactions in response to Sputnik V (Gam-COVID-Vac) vaccine were longer (up to 3 days) than reactions in response to Sinopharm vaccine (a few hours) [30]. The frequency of post-vaccination reactions is also varied with at least one reaction occurring in 93.2 % cases of Sputnik V (Gam-COVID-Vac) vaccine users and 87.3 % of Sinopharm (BBIBP-CorV) vaccine users. The most frequent reactions were pain at the injection site, headache and malaise. Furthermore, in the study there were cases of thrombosis and conditions related to clotting disorders: 7 cases after administration of Sputnik V (Gam-COVID-Vac) vaccine and 3 cases after administration of Sinopharm (BBIBP-CorV) vaccine. However, a subsequent association between vaccination and conditions related to clotting disorders has not been found for both vaccines, and this issue requires further investigation [30]. Another study based on a vaccines survey of the vaccinated with Sputnik V (Gam-COVID-Vac), Sinopharm in Iran [31], aimed to investigate cutaneous reactions in response to the administration of Sputnik V (Gam-COVID-Vac) and Sinopharm vaccines. Skin manifestations in the vaccinated people were transient. The most common reactions were pain in the injection site (79.9 % of Sputnik V (Gam-COVID-Vac) vaccine users, 60.6 % of Sinopharm vaccine users), tightening of the injection site (13.4 % of Sputnik V (Gam-COVID-Vac) vaccine users, 10.3 % of Sinopharm (BBIBP-CorV) vaccine users), reddening of the skin at the injection site (12.8 % of Sputnik V (Gam-COVID-Vac) vaccine users, 6.9 % of Sinopharm (BBIBP-CorV) vaccine users) [31].

### 5.4 Implications for practice or policy

The sample sizes of available international publications directly comparing the immunological efficacy and reactogenicity of Sputnik V (Gam-COVID-Vac) and Sinopharm (BBIBP-CorV) vaccines still leave a knowledge gap.

### 5.5 Findings of the present study in light of what was published before

However, there are a few publications suggesting that vaccinated people with prior infection are most protected against COVID-19 [32,33]. Fig. 6 in our study also proves this trend based on the obtained immunogenicity data. Therefore, seropositive patients with antibodies after a natural course of infection will have a long-lasting and much more intense immune response to vaccination both with Sputnik-V and Sinovac vaccines (hybrid immunity).

### 5.6 Strengths and limitations of the study

Several limitations should be considered while interpreting our manuscript: sample size, limited age of participants (from 29 to 73 years), lack of an orthogonal immunoassay to confirm the results obtained from the utilized kit, lack of neutralizing antibodies and T-cell response assessments. It's necessary to emphasize that this study is still underpowered to detect rare side effects, what may be done only in larger multicenter post-registration studies. Nevertheless, the strength and clinical importance of our study is in the direct comparison of Sputnik V and Sinopharm in real-life settings. Among the strong points of this study are simultaneous comparison of 2 different vaccines in the same epidemiological conditions (the same circulating SARS-CoV-2 strains) and the double-controlled system of data collection, which helped to avoid the missing data in the analysis.

## 6 Conclusions

In conclusion, the Sputnik V (Gam-COVID-Vac) vaccine has higher immunogenicity rates on the 42nd day and in 6 months after the first dose compared to Sinopharm (BBIBP-CorV). This trend was seen among both groups previously infected with SARS-CoV-2 and those without a history of COVID-19. This study also confirmed that a combination of post-vaccination antibodies and those developed as a result of prior SARS-CoV-2 infection creates a higher levels of antibodies, which persist for a longer time both in Sputnik V and Sinopharm study groups. In a multivariate analysis, IgG seropositivity at a baseline and Sputnik V vaccine type were shown to be significantly associated with higher IgG levels both at 42 days and at 6 months post-vaccination.

The main reactions after vaccination were fever, redness and soreness in the injection site. The reactogenicity of both vaccines was relatively similar, while a fever after vaccination was more common among those vaccinated with Sputnik V (Gam-COVID-Vac) compared to vaccinated with Sinopharm (BBIBP-CorV) vaccine.

Authors have reported no relevant conflict of interest regarding to this study exist.

## Declaration of competing interest

The authors declare that they have no known competing financial interests or personal relationships that could have appeared to influence the work reported in this paper.

## Figures and Tables

**figs1:**
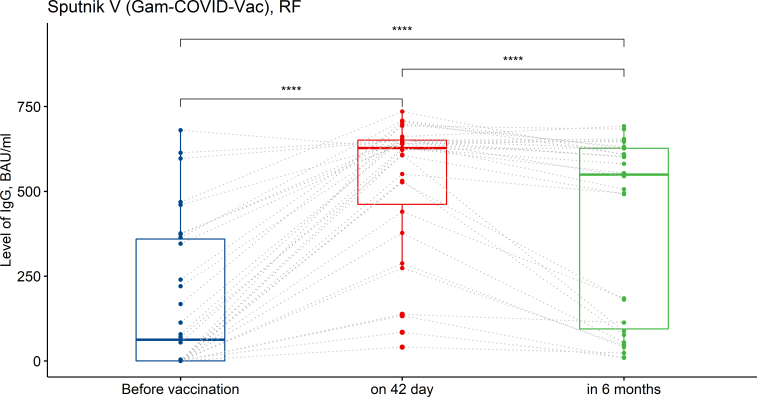


**figs2:**